# Surgical Outcomes, Ocular Safety and Tolerability of Bio-Interventional Cyclodialysis with Allograft Scleral Reinforcement: Clinical Experience of More than 240 Cases

**DOI:** 10.3390/jcm13164593

**Published:** 2024-08-06

**Authors:** Craig J. Chaya, Leon W. Herndon, Jorge Lince, Nathan Radcliffe, Ehsan Sadri, Arkadiy Yadgarov, Tsontcho Ianchulev

**Affiliations:** 1 Department of Ophthalmology and Visual Sciences, Moran Eye Center, University of Utah, Salt Lake City, UT 84112, USA; 2 Department of Ophthalmology, Duke Eye Center, Duke University, Durham, NC 27708, USA; 3 Department of Ophthalmology, New York Eye and Ear of Mount Sinai, New York, NY 10003, USA; 4 Visionary Eye Institute, Newport Beach, CA 92663, USA; 5 Coastal Research Associates, Atlanta, GA 30076, USA

**Keywords:** glaucoma, scleral allograft, cyclodialysis, supraciliary space

## Abstract

**Background:** To report the surgical safety of reinforced bio-interventional cyclodialysis with scleral allograft reinforcement. **Methods:** This was a consecutive case series of 243 eyes with open-angle glaucoma who underwent a bio-scaffolded cyclodialysis (BSC) procedure for uveoscleral outflow enhancement using allogeneic bio-spacers to maintain patency of the internal filtration conduit. **Results:** 79% of the eyes underwent concomitant phacoemulsification cataract surgery prior to BSC intervention, while the remaining eyes underwent stand-alone BSC surgery. All patients had a postoperative surgical safety period of at least 30 days. There were no sight-threatening or serious ocular adverse events. There was one case of prolonged iritis beyond 30 days, which resolved with topical treatment. Two cases (0.8%) of intraoperative and five (2%) of postoperative non-sight-threatening hyphema were without clinical sequelae, which resolved with conservative management. There were 11 cases of IOP elevation and one case of numeric hypotony without maculopathy, which resolved within the study period. The rate of secondary surgical intervention for IOP control was low, and overall, IOP for the cohort improved in the postoperative period, with 78.6% of eyes achieving IOP ≤ 18 mmHg without an increase in medications. **Conclusions:** Allogeneic biotissue for cyclodialysis intervention demonstrates a biocompatible ocular profile as an implantable material for internal scleral reinforcement during uveoscleral outflow enhancement surgery.

## 1. Background

Scleral allograft tissue has a long-standing clinical use in ocular surgery. Conventional application over the last several decades has been mostly for ab-externo scleral reinforcement of the episcleral/subconjunctival space as an adjunct to glaucoma drainage devices to prevent tube erosion [1,2,3,4]. It is also used for reinforcement and patch grafting/reconstruction of scleral defects, such as those occurring during episodes of necrotizing scleritis [5]. In all these cases, the scleral allograft has demonstrated excellent safety as an inert biocompatible and non-biodegradable material that can reinforce the ocular surface and the integrity of the scleral interface/wall, as well as protect the ocular tissues from the erosive effects of implantable hardware, such as glaucoma drainage devices.

More recently, interventional ab-interno delivery techniques have opened new applications for scleral allograft tissue [6]. The acellular scleral matrix, when minimally modified and delivered with ab-interno micro-interventional instrumentation, can provide durable and non-resorbable structural support for glaucoma and retina surgical applications. The biomaterial properties of the scleral allograft can enable both conductive and occlusive ab-interno stenting and reinforcement. One example from the surgical retina is the management of optic disc pit maculopathy and endoscleral closure of scleral defects [7,8,9]. In a case series, ab-interno scleral allograft reinforcement has been shown to be well-tolerated without any complications in the posterior segment when implanted into the optic disc pit to occlude subretinal cerebrospinal fluid leakage and resolve fluid accumulation. In surgical glaucoma, intraocular implantation of a scleral allograft in the supraciliary space has been shown to structurally enhance and maintain suprachoroidal outflow because of the reinforcing properties of the highly permeable and porous allograft acellular matrix [10].

Scleral allograft has important properties which make it useful and desirable as a biotissue substrate for homologous structural reinforcement, stabilization, reconstruction, and stenting. The sclera is acellular and can be gamma-sterilized for a long shelf life (up to 2 years) in standardized packaging [11]. This is important, as acellular tissue is deprived of antigen-presenting cells and has a low immunogenic profile, which is further enhanced by the sterilization process [12,13]. Allograft is non-biodegradable and has high structural integrity, a high tensile strength, and non-brittle, leathery physical properties, making it remarkably resilient and an ideal substrate for structural reinforcement [14,15]. Its flexibility and conformability enable a wide range of surface applications when used to patch grafts of a scleral wall defect.

In addition, the sclera is highly permeable and hydrophilic, with important properties when it comes to aqueous conductivity [16,17,18].

**Figure 1:**
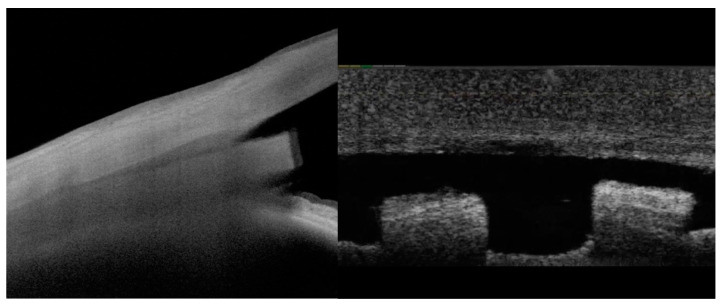
Scleral allograft bio-reinforcement in the eye demonstrates high homogeneity with native tissues on OCT imaging. High-resolution longitudinal OCT imaging using Anterion^®^ OCT and transverse OCT imaging of the allograft at the endoscleral (supraciliary) interface.

When used for endoscleral or episcleral reinforcement, the allograft implant is homologous and has biological and biomechanical properties similar to those of the native surrounding tissue (Figure 1). This is important regarding the risk of implant fibrosis, as sterilized allograft scleral tissue has one of the lowest indices of material stiffness mismatch when implanted adjacent to the scleral surface, thereby reducing the potential for fibrosis and macrophage activation as compared to stiffer implantable non-biologic materials such as nitinol or polyimide [19,20].

**Figure 2:**
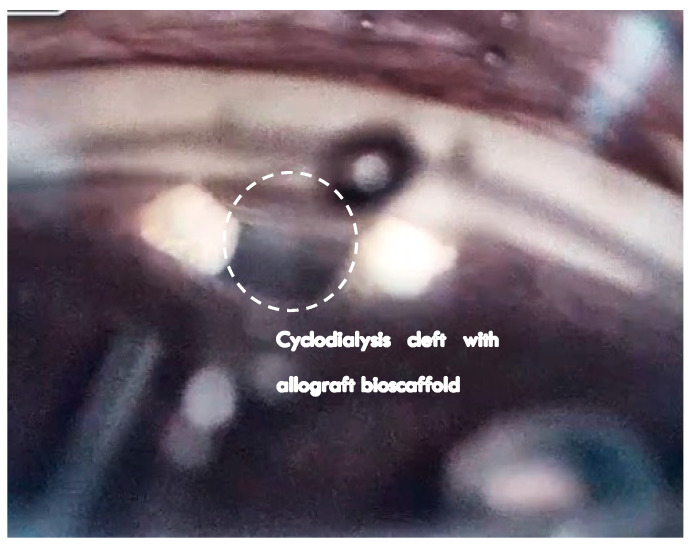
In bio-scaffolded cyclodialysis (BSC), a cyclodialysis cleft is formed, and cuboidal allograft spacers are placed laterally to reinforce the central filtration channel, allowing for enhanced aqueous outflow through the cleft.

In this interventional series, acellular homologous allograft biotissue is used for scleral reinforcement of the endoscleral wall of a cyclodialysis. Such bio-interventional reinforcement and scaffolding of the cyclodialysis cleft is designed to enhance the internal filtration reservoir and create a durable conduit for uveoscleral outflow conductivity (Figure 2). Cyclodialysis has long been used as an internal aqueous filtration channel to the suprachoroidal space and is effective in lowering the IOP in glaucoma patients [21,22,23,24,25]. In some patients, the durability of the effect is limited due to premature closure of the cleft or attenuation of the filtration conduit. Implantable hardware and stenting devices have been increasingly used to maintain the durable patency of cyclodialysis and internal uveoscleral filtration channels. Major randomized controlled clinical trials demonstrate long-term IOP lowering with supraciliary stents and MIGS devices [26,27,28], although the mechanical properties of the rigid implantable hardware materials have been shown to impact the surrounding tissues and cause foreign-body reactions.

In the BSC procedure, flexible allogeneic bio-scaffolding is deployed homologously within the cyclodialysis at the endoscleral wall for reinforcement and structural stabilization of the filtration cleft. The allograft bio-scaffolding matrix is acellular, non-resorbable, porous, and hydroconductive—all highly desirable biomaterial features for ab-interno intraocular applications. Unlike suprachoroidal aqueous drainage devices [26,27,28], bio-scaffolding reinforces internal filtration through the native cyclodialysis reservoir, which drives the primary aqueous outflow enhancement.

Herein, we report the results of the largest clinical series of intraocular allogeneic biotissue implantation.

## 2. Methods

This interventional case series was pooled from the CREST US and CREST OUS observational real-world evidence registries, which comprise the entire available clinical study experience to date for ab-interno scleral allograft implantation for cyclodialysis reinforcement (registration number: NCT05506423). The CREST US and OUS registries are multi-center studies designed to follow patients who underwent interventional cyclodialysis with supraciliary implantation of an allograft scleral biotissue for reinforcement of the cyclodialysis cleft. All patient data were anonymized and treated with confidentiality according to the tenets of the Declaration of Helsinki, and institutional review board approval was obtained for all study sites. The current dataset comprises all 9 surgical sites with evaluable safety follow-up of at least 30 days postoperatively.

### 2.1. Eligibility Criteria

Patients enrolled in the CREST had a confirmed diagnosis of open-angle glaucoma (OAG). To be eligible for the study, patients must have undergone a BSC procedure using the CycloPen micro-interventional system (Iantrek, Inc., White Plains, NY, USA). Inclusion criteria for the registry were the presence of open-angle glaucoma, which required clinical diagnostic confirmation with Schaeffer Grade >2 angles, age greater than 18 years, and ability to sign an informed consent. Exclusion criteria were narrow-angle glaucoma, narrow anatomic angles, prior laser or surgical iridotomy, axial length >26.0 mm, and active ocular inflammation.

### 2.2. Implantation Technique and Material

The allograft implant was prepared and processed from a standard sterile donor scleral patch graft supplied by an eye bank. The tissue was micro-trephined using high-precision AlloFine™ (Iantrek, Inc., White Plains, NY, USA) instrumentation and process to create a micro-scaffold of 500 microns width and 5 mm length for endo-scleral reinforcement. The bio-scaffold was then loaded into a cannulated carrier coupled to a micro-interventional cyclodialysis delivery system (CycloPen ™, Iantrek, Inc., White Plains, NY, USA) for ab-interno deployment of the biotissue.

**Figure 3:**
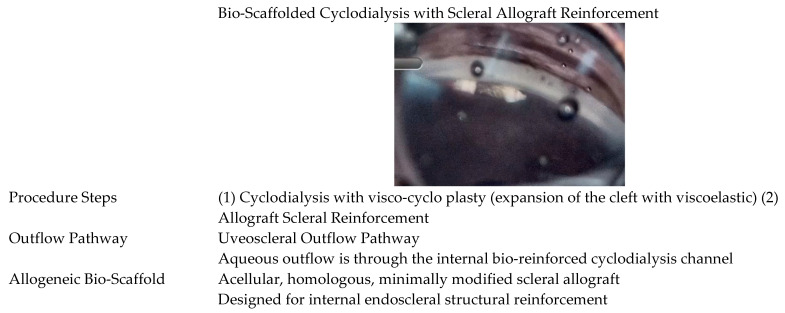

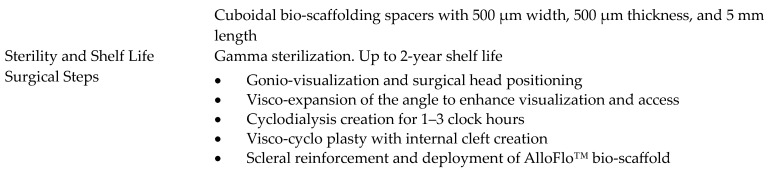
Allogeneic Bio-Scaffolded Cyclodialysis with Scleral Allograft Reinforcement.

The surgical technique comprised a two-step sequential intervention, which was performed using standard gonioscopic visualization and positioning. The first step of the procedure was cyclodialysis. Using a cyclodialysis cannula or spatula, a controlled focal ciliary body disinsertion of 1–3 clock hours was constructed up to 5 mm posterior to the limbus in the nasal quadrant. Visco-cyclo pasty with a dispersive or cohesive viscoelastic was then performed to expand the cleft prior to scleral reinforcement. The second step of the multi-interventional procedure was scleral reinforcement. The allograft bio-scaffolding was implanted at the endoscleral surface above the ciliary body using the CycloPen delivery system. The ab-interno scleral reinforcement extended 5 mm posteriorly to maintain the entire depth of the cleft and enhance the structural stability of the uveoscleral filtration channel. Gonioscopic confirmation ensured proper biotissue deployment within the cyclodialysis cleft flush with the iris root (Figure 2 and Figure 3).

When in combination with cataract surgery, the phacoemulsification procedure was completed first, and the intraocular lens was implanted. All cases underwent standard phacoemulsification cataract extraction and IOL implantation using the surgeon’s phaco technique. There were no modifications to any steps of the surgeon’s preferred phaco procedure related to the preceding glaucoma surgery. Safety was assessed in a pooled cohort and separately in eyes where cyclodialysis with scleral reinforcement was performed with or without cataract surgery.

### 2.3. Data Analysis

Descriptive statistics were used to characterize ocular safety and tolerability and the rate of AEs and SAEs. Differences in continuous data are expressed as mean ± standard deviation (SD) and compared using paired two-tailed *t*-tests. Categorical data are presented as numbers and percentages and were compared where indicated using the Chi-square test. Statistical significance was defined as *p* < 0.05. Statistical and graphing software included Excel 16.87 (Microsoft Corp., Redmond, WA, USA) and Prism v.9.0 (GraphPad Software, Boston, MA, USA).

## 3. Results

A total of 245 consecutive eyes were enrolled in this interventional registry and underwent uveoscleral outflow enhancement using a two-step procedure of cyclodialysis and allograft scleral reinforcement. Of these, two eyes were not implanted, with one due to anesthesia concerns and another due to a deployment device malfunction, leaving 243 interventional cases. At month 1 (M1), nine eyes were either lost-to-follow-up (n = 5), missed the month 1 visit (n = 2), or were withdrawn prior to the M1 visit due to study-nonadherence to registry requirements, leaving 234 eyes evaluable at M1 (Table 1).

Depending on the size of the bio-scaffolded cyclodialysis, the results were stratified and evaluated in two subgroups: Group 1 (n = 153, 63.0%), where the size of the bio-reinforced cyclodialysis was less than 1 clock hour, and Group 2 (n = 90, 37.0%), with eyes with cyclodialysis greater than 1 clock hour. The baseline characteristics were similar between the two groups (Table 2). Group 2 had a higher rate of phaco-combined intervention (87.6% vs. 64.4%) with a higher medication burden at baseline (1.7 vs. 1.3 average number of medications).

Intraoperative safety was unremarkable (Table 3). There were no clinically significant vision-threatening adverse events. Mild supraciliary blood reflux during the procedure is expected in most gonio-interventional procedures. More significant intraoperative hyphema requiring visco-tamponade was reported in two cases, and both resolved. There was one case of zonular dehiscence due to patient eye movement during the irrigation/aspiration portion of the phaco procedure.

Postoperative safety was characteristic of supraciliary intervention. There was one serious adverse event (AE) reported (pneumonia with hospitalization) deemed unrelated to the ocular surgery. In the early follow-up period of 1 week, there were five cases of hyphema >2 mm, all of which were resolved with conservative management before the month 1 visit. There was one case of asymptomatic macular folds with no clinical hypotony maculopathy in the setting of persistent iritis, which was managed and resolved without visual sequelae. There was one case of transient iritis beyond the 30-day postoperative period, which was resolved with conservative management. There was one case of clinically significant hypotony, which resolved within the first month postoperatively. There was one phaco surgery-related postoperative complication of retained cortical segment removal (Table 3).

There were 11 eyes with postoperative IOP elevation > 30 mmHg and/or an increase of 10 mmHg from baseline. Three eyes underwent additional surgical interventions for IOP control. One case underwent a paracentesis 2 weeks after surgery. Another case received an XEN gel implant approximately 2 months postoperatively. The third case was managed with YAG laser treatment to disrupt the Gonio-synechiae around with additional selective laser trabeculoplasty. The remainder of the eight eyes with elevated IOP were managed with topical hypotensive medications.

There were four eyes of three patients who developed cystoid macular edema (CME) in the setting of a concurrent phaco procedure, which was not attributable by investigators to glaucoma intervention. All of these were transient, not associated with significant vision loss, and resolved completely in the postoperative period. In one, the CME was in the setting of pre-proliferative diabetic retinopathy. The single patient with a macular fold was in the setting of uneventful cataract and glaucoma surgery with a postoperative IOP that was always above 7 mmHg and was associated with transient iritis, which recovered with topical non-steroidal anti-inflammatory treatment. The event was not associated with macular edema and did not result in a decrease in visual acuity, achieving full resolution during the study follow-up period.

Of all evaluable subjects, best-corrected distance visual acuity was either maintained or improved in the case with combined phacoemulsification, and there were no AEs or incidence of significant, >2 lines BDCVA loss postoperatively in relation to the surgery. Residual postoperative refractive error > −1.00 D occurred in seven cases (2.9%).

Intraocular pressure (IOP) outcomes showed improvement, with statistically significant reductions in all groups. Mean (SD) medicated IOP in Groups 1, 2, and All Eyes improved from 20.4 (5.5), 18.9 (6.2) and 19.8 (5.8) at baseline to 14.8 (5.4), 13.2 (4.2) and 14.2 (5.0) mmHg at 30 days, respectively, with statistical significance of *p* < 0.001 in each group. Similarly, the proportion of eyes with IOP at or below 18 mmHg improved for all Group 1, Group 2, and All Eyes from 43.8%, 50.0%, and 46.1% at baseline to 75.7%, 83.7%, and 78.6% at 30 days, respectively, with a statistical significance of *p* < 0.001.

There were 207 eyes with postoperative refractive outcomes from the cataract procedure in the registry. There were only seven cases (3.3%) with refractive error exceeding −1.00 D postoperatively.

## 4. Discussion

We report the surgical outcomes of more than 200 cases of scleral allograft implantation for intraocular ab-interno endoscleral reinforcement. In addition, our interventional series provides extensive clinical experience with interventional cyclodialysis for the enhancement of uveoscleral outflow.

There is a paucity of data on the clinical performance and safety of scleral allograft for intraocular implantation and internal reinforcement. Our results show a low rate of intraoperative and postoperative complications with a safety profile characteristic of a well-tolerated, biocompatible material. There was minimal postoperative iritis and intraocular inflammation, with good implant stability and no migration. There was no acute reaction to the allogeneic biotissue, which is acellular and homologous to the surrounding native tissues. Slit lamp bio-microscopy of the anterior chamber is one of the more sensitive and quantifiable approaches to evaluate the inflammatory response in the body at a cellular level, and it is encouraging that the incidence of postoperative inflammation and iritis are indistinctly similar to the standard-of-care phaco procedures. In addition, we see evidence of early post-implantation stability and no apparent postoperative migration, which is likely a result of the non-lubricious biomechanical textured surface of the allograft and its flexible, bio-conforming nature. This clinical profile of the allograft when implanted ab-interno is similar to the clinical experience from ab-externo scleral reinforcement, where the allograft tissue is used on the episcleral surface with minimal reaction, rejection and long-lasting durability [6].

In addition, our study is informative on the safety of ab-interno cyclodialysis—a procedure with long-standing historical use in ophthalmology for IOP lowering [23,24,29,30]. Unlike conventional cyclodialysis procedures, the interventional approach used was minimally invasive, with only 1–3 clock hours of focal supraciliary cleft dissection. Besides the minimal reflux expected from gonio-interventional MIGS procedures, there was a very low rate of clinical sequelae, such as postoperative hyphema and hypotony, all of which were transient and resolved with conservative follow-up. Also, the sequential allograft bio-reinforcement of the cyclodialysis was anatomically successful in all cases with early postoperative structural stability and a safety and functional IOP effect.

Longer observation and follow-up are needed to determine the long-term efficacy and durability of the procedure. Prior attempts at maintaining the patency of cyclodialysis by using transient cleft maintainers such as air or viscoelastic have met with limited success [21,23]. Whether scleral allograft reinforcement can address early closure of the cleft in some patients and maintain long-term patency of the uveoscleral outflow channel remains to be seen in long-term studies. Similarly, implantable scleral allograft can mitigate some of the issues of implantable hardware in the suprachoroidal space, which were seen with rigid non-biologic devices such as CyPass (Alcon, Ft. Worth, TX, USA) or the iStent Supra (Glaukos, Aliso Viejo, CA, USA). The highly porous, hydrophilic, and permeable scleral allograft offers aqueous conductivity and wicking drainage posteriorly in the suprachoroidal space, similar to what has been seen with other conductive non-cannulated implants such as the MINIject (iStar, Inc., Wavre, Belgium). How these biomaterial properties translate into long-term clinical benefits remains the subject of additional investigations.

Our study demonstrates that the glaucoma procedure of bio-reinforced cyclodialysis can be successfully combined with cataract surgery—in fact, the majority of the patients in our cohort underwent combined phaco-glaucoma intervention for mild-moderate glaucoma patients with cataract co-morbidity. There was no untoward effect of the glaucoma procedure on cataract surgery. From a refractive standpoint, the incidence of residual postoperative refractive error exceeding −1.00 D postoperative was relatively low at only 2.9%. This is consistent with other studies of refractive outcomes from cataract surgery where similar rates of 6–7% have been reported from large population health surveys, some exceeding 250,000 patients with a rate of 85% of subjects achieving refractive outcomes within 1.00 diopters spherical equivalent of the target [31,32,33]. This indicates that there is no appreciable signal of myopic shift after the bio-reinforced cyclodialysis intervention.

This is a real-world clinical evidence study of consecutive cases and represents the largest cohort of implantable scleral biotissue to date. The number of cases is informative from a safety standpoint and demonstrates a high degree of tolerability and ocular safety of homologous, sterile, and minimally manipulated implantable scleral allograft tissue. It is encouraging to see no emergent safety events for what appears to be a biocompatible acellular matrix that can be applied for structural reinforcement and scaffolding inside the eye. In addition, cyclodialysis intervention with bio-reinforcement has the benefit of an ab-interno approach utilizing a reservoir space internal to the eye for IOP lowering, thus avoiding serious complications such as hypotony, endophthalmitis, bleb infection, or leaks as seen with conventional penetrating bleb-forming glaucoma surgery such as trabeculectomy and tubes.

This study intended to report the intraoperative and early postoperative safety and surgical feasibility and early outcomes of cyclodialysis with a supraciliary reservoir using implantable allograft scaffolding. With this intent, its design is limited, focusing mostly on the immediate postoperative period up to 30 days post-op. Certainly, long-term follow-up with extended safety and efficacy outcomes is needed.

## Figures and Tables

**Table 1:** Subject Accountability.

Total eyes with BSC intervention	243
Lost-to-follow up	5
Missed M1 visit	2
Withdrew from study	2
Total eyes evaluable at 30 days post-op	234

**Table 2:** Baseline Demographics.

	Group 1 ≤1 Clock h Cyclodialysis	Group 2 >1 Clock h Cyclodialysis	All Eyes
Eyes, N	153	90	243
Eyes evaluable at 30 days (M1), N	148	86	234
Age, Mean ± SD (years)	72.2 ± 8.2	70.9 ± 8.2	71.7 ± 8.2
Female, N (%)	71 (46.4%)	51 (56.7%)	122 (50.2%)
Race/Ethnicity			
White	56 (36.6%)	10 (11.1%)	66 (27.2%)
Black or African American	21 (13.7%)	35 (38.9%)	56 (23.0%)
Hispanic or Latin American	75 (49.0%)	43 (47.8%)	118 (48.6%)
Asian	1 (0.7%)	2 (2.2%)	3 (1.2%)
Baseline BCVA (decimal)	0.46 ± 0.31	0.52 ± 0.27	0.48 ± 0.29
Number of cases combined with phaco, N (%)	134 (87.6%)	58 (64.4%)	192 (79.0%)
B/L IOP lowering medications (Mean ± SD)	1.3 ± 1.0	1.7 ± 1.5	1.5 ± 1.2
B/L medicated IOP (Mean ± SD)	20.4 ± 5.5	18.9 ± 6.2	19.8 ± 5.8

B/L = Baseline, IOP = Intraocular pressure, SD = standard deviation, BCVA = best-corrected visual acuity.

**Table 3:** Safety Outcomes (N = 245).

**Intraoperative**	
Inability to deploy implant, n (%)	1 (0.4%)
Anesthesia reaction (preventing implant deployment), n (%)	1 (0.4%)
Zonular dialysis during phacoemulsification, n (%)	1 (0.4%)
Intraoperative hyphema/reflux, n (%)	2 (0.8%)
**Postoperative**	
Pneumonia/hospitalization, n (%)	1 (0.4%)
Hypotony, transient, n (%)	1 (0.4%)
IOP elevation (>30 mmHg or +10 mmHg from baseline), n (%)	11 (4.4%)
Post-op hyphema >2 mm present after 1 day post-op, n (%)	5 (2.0%)
Macular edema, cystoid n (%)	4 (1.6%)
Macular folds, anatomic, no maculopathy: no visual sequelae	1 (0.4%)
Iritis transient (unrelated to biotissue implantation), n (%)	1 (0.4%)
Additional laser intervention	2 (0.8%)
YAG laser (synechiae)	1 (0.4%)
Selective laser trabeculoplasty (SLT)	1 (0.4%)
Additional surgical intervention (glaucoma)	3 (1.2%)
Corneal wound burp	1 (0.4%)
Paracentesis	1 (0.4%)
XEN gel stent	1 (0.4%)
Additional surgical intervention (cataract)	
Cortical remnant removal	1 (0.4%)

## Data Availability

All data generated or analyzed during this study are included in the body of this published article. Raw data are available upon reasonable request.

## Abbreviations

Registry study of CycloPen Micro-Interventional System and Long-Term Clinical Outcomes (CREST), bio-scaffolded cyclodialysis (BSC), adverse event (AE), severe adverse event (SAE), intraocular pressure (IOP), selective laser trabeculoplasty (SLT), optical coherence tomography (OCT), open-angle glaucoma (OAG), cystoid macular edema (CME), best-corrected distance visual acuity (BCDVA).
